# Incorrect Address

**DOI:** 10.1001/jamahealthforum.2022.4425

**Published:** 2022-11-11

In the JAMA Forum titled “Health Insurance Coverage After the COVID-19 Public Health Emergency Ends,”^1^ published online September 29, 2022, in *JAMA Health Forum*, the corresponding author’s address was incorrect. In the corresponding author section, the address should have been “1220 19th St NW, Washington, DC 20036.” This article was corrected online.
